# Conditioned Medium from H_2_O_2_-Preconditioned Human Adipose-Derived Stem Cells Ameliorates UVB-Induced Damage to Human Dermal Fibroblasts

**DOI:** 10.3390/antiox11102011

**Published:** 2022-10-11

**Authors:** María Burón, Teodoro Palomares, Patricia Garrido-Pascual, Borja Herrero de la Parte, Ignacio García-Alonso, Ana Alonso-Varona

**Affiliations:** 1 Department of Cell Biology and Histology, Faculty of Medicine and Nursing, University of the Basque Country (UPV/EHU), 48940 Leioa, Bizkaia, Spain; 2 Department of Surgery, Radiology and Physical Medicine, Faculty of Medicine and Nursing, University of the Basque Country (UPV/EHU), 48940 Leioa, Bizkaia, Spain

**Keywords:** photodamage, oxidative stress, human adipose-derived stem cells, dermal fibroblasts, H_2_O_2_-preconditioning, ultraviolet B radiation, cell therapy

## Abstract

Human skin exposure to ultraviolet B (UVB) radiation can result in acute photodamage through oxidative modifications of cellular components and biomolecules involved in the metabolism of dermal cells. Recently, the therapeutic potential of human adipose-derived stem cells (hASCs) has been investigated as a novel strategy for photoprotection due to their pro-angiogenic properties, protective activity against oxidative stress and paracrine effect on dermal cells. To enhance these therapeutic properties, hASCs can be preconditioned by exposing them to sublethal cellular stressors. In this study, we first analyzed response capacity against UVB-induced oxidative stress in H_2_O_2_-preconditioned hASCs (called HC016 cells); and second, we evaluated the photoprotective effect of HC016-conditioned medium (CM) in an in vitro UVB irradiation model in cultured human foreskin fibroblasts (hFFs). The results demonstrated that HC016 cells have a greater capacity to respond efficiently to UVB-induced oxidative stress, evidenced by higher Nrf2 antioxidant system activity and enhanced viability and migration capacity. Further, HC016-CM treatment increased viability, migratory capacity and collagen type I synthesis in hFFs exposed to UVB radiation, as well as reducing their cytotoxicity, apoptosis, senescence and IL-6 secretion. Collectively, these findings support the view that HC016 cells could protect against UVB-induced photodamage via paracrine mechanisms.

## 1. Introduction

Human skin exposure to ultraviolet B (UVB) radiation can result in acute photodamage through oxidative modifications of cellular components and biomolecules involved in the metabolism of dermal cells. As a result, there is disruption of cell function and skin disorders may develop [1,2,3], including allergy disorders, UV-increased sensitivity by drugs, photoaging and carcinogenesis [4,5]. UVB is mostly absorbed in the epidermis and mainly affects epidermal cells, but 10–30% of UVB can penetrate the epidermis and damage dermal fibroblasts, reducing their metabolic activity and synthesis of elastic and collagen fibers, in turn, inducing expression of senescence-associated β-galactosidase (SA-β-Gal) and reducing their migration activity [6,7,8].

From a molecular point of view, UVB radiation damage to dermal fibroblasts occurs both via direct DNA harm and indirectly through the formation of reactive oxygen species (ROS), resulting in an alteration of redox homeostasis and skin inflammation [9,10,11]. Oxidative stress can disrupt the homeostasis of skin cells through damage to the DNA and proteins, and also interfere with cell signaling pathways leading to cell death, apoptosis or malignant transformation [5,12]. This redox imbalance affects the redox-sensitive nuclear factor erythroid 2-related factor 2 (Nrf2), which is negatively regulated by Kelch-like ECH associated protein 1 (Keap1) [13]. In basal conditions, the Nrf2-Keap1 complex is localized in the cytosol, but under conditions of redox imbalance, the complex dissociates and the released Nrf2 translocates into the nucleus [14,15]. Nrf2 protein, through interaction with DNA, regulates the expression of antioxidant response element (ARE), which is important for protection against oxidative damage [16]. In addition, UVB irradiation induces the activation of interleukin (IL)-1, epidermal growth factor (EGF) and tumor necrosis factor (TNF) receptors, which leads to the transcription of the activator protein 1 (AP-1) and nuclear factor kappa B (NF-kB), increasing the expression of matrix metalloproteinases (MMPs) and collagen degradation [17,18].

Currently, there are many approaches to preventing or treating skin photodamage, including optical therapies, fibroblast/cytokine injection, and the application of sunscreen, antioxidants and other topical products; however, many of these strategies can alter the skin’s endocrine function and produce harmful effects, such as allergies, phototoxic reactions or irritations [19]. Therefore, potential therapy with mesenchymal stem cells (MSCs) has aroused considerable interest due to their pro-angiogenic properties, protective activity against oxidative stress and paracrine effect on dermal cells [20]. Specifically, MSCs can act directly by migrating towards the injured sites and differentiating into target tissues, or through paracrine mechanisms [21]. The production and secretion of soluble factors via the secretome is an intrinsic characteristic of MSCs, which provides cues necessary for the restoration of injured tissues [22,23]. These soluble factors include growth factors and cytokines, which have been suggested to possibly protect fibroblasts from oxidative stress, reducing apoptosis, improving their survival and promoting type 1 procollagen synthesis [24,25].

Although it is possible to obtain MSCs from various niches, human adipose-derived stem cells (hASCs) offer a number of advantages over stem cells from other sources. In particular, hASCs are found in abundant quantities and they are harvested by a minimally invasive procedure, can differentiate into multiple cell lineages in a regulated and reproducible manner, and are safely transplanted in both autologous and allogeneic ways [26]. Several preconditioning strategies have already been used to improve therapeutic properties of hASCs [27,28]. One of them, sublethal exposure to cellular stressors, induces the expression and secretion of molecules that reduce cell damage and increase cell survival. This allows cells to respond efficiently to a higher level of the same stressor [29,30]. In previous studies, we demonstrated that preconditioning of the hASCs with low doses of H_2_O_2_ (so-called HC016 cells) endows them with increased resistance to oxidative stress. It is mainly due to an increase in their antioxidant capacity and bioenergetic regulation [31,32]. Furthermore, we showed that HC016-conditioned medium (CM) also increases their capacity to repair oxidative damage suffered by other cell lines, specifically in an in vitro model of oligodendrocyte-like cells [33].

Since one of the main action mechanisms in UVB-induced damage is oxidative stress, the aim of this study was to investigate the photoprotective effect of HC016-CM, in an in vitro UVB irradiation model in cultured human foreskin fibroblasts (hFFs). For this purpose, we analyzed the harmful effect of UVB radiation on hFFs at both cellular and molecular levels.

## 2. Materials and Methods

### 2.1. Cell Culture

Both the hASCs and hFFs used in this study were courtesy of Histocell S.L. (Derio, Spain). Cell cultures were maintained, and then expanded up to passage 4 in Dulbecco’s Modified Eagle Medium Glutamax™ (DMEM, Gibco, Paisley, UK) supplemented with gentamycin (1 µL/mL, Sigma-Aldrich, St. Louis, MO, USA) and 10% heat-inactivated fetal bovine serum (FBS, Biochrom, Berlin, Germany). Incubator environmental conditions were set at 37 °C, humidified atmosphere and 5% CO_2_.

### 2.2. H_2_O_2_-Preconditioning of hASCs

H_2_O_2_-preconditioned hASCs (HC016 cells) were obtained by long-term exposure to a low concentration of H_2_O_2_ (PanReac AppliChem, Barcelona, Spain). Briefly, hASCs were exposed to 10 µM of H_2_O_2_ for 7 days, replacing the oxidative culture medium every 3–4 days (see details in HC016 patent; WO/2013/004859, 2013). Simultaneously, hASCs were carried out in the standard medium as described in the previous section. Once preconditioning was completed, both HC016 cells and hASCs were seeded at high density in complete medium for 18–20 h at 37 °C and incubated in a humidified atmosphere containing 5% CO_2_ until use.

### 2.3. Ultraviolet B Irradiation

At 24 h after plating, cells were washed twice with 1× PBS and exposed to UVB radiation in a thin layer of PBS, using a UVLM-26 lamp (Ultra-Violet Products Ltd., Cambridge, UK), emitting at 302 nm. Immediately after the UVB irradiation, the PBS was aspirated and replaced with FBS-free DMEM, and cultures were incubated for the times established for each experiment.

The initial UVB broadband doses tested were 10 to 80 mJ/cm^2^_,_ and three doses below the 50% lethal dose (LD_50_) were chosen for ensuing experiments. Control cells were kept in the same culture conditions without UVB exposure.

### 2.4. hFF Treatment with HC016-Conditioned Medium

To obtain HC016-CM, HC016 cells were grown in T175 (Sarstedt, Nümbrecht, Germany) tissue culture flasks to 80% confluence, washed three times with 1× PBS and incubated for 48 h in FBS-free DMEM Glutamax™. Then, the CM was centrifuged for 5 min at 250× *g*, filtered through a 0.2-µm filter (Filtropur S 0.2, Sarstedt, Nümbrecht, Germany) and stored at −20 °C until use.

To evaluate the therapeutic potential of HC016-CM, before UVB irradiation, the hFFs were cultured for 24 h with the CM at 37 °C, in a humidified 5% CO_2_ atmosphere (CM+ hFFs). After that period, cells were irradiated as previously described, and PBS was replaced with fresh FBS-free DMEM. Control cells were kept under the same culture conditions without CM treatment (CM− hFFs).

### 2.5. Proliferation Assays

Cell proliferation was determined in 96-well plates (Sarstedt, Nümbrecht, Germany) containing 10^4^ cells/well. PrestoBlue^®^ reagent (Invitrogen, Eugene, OR, USA) was used to assess cell viability at 24 and 48 h. Procedure was performed according to manufacturer’s instructions measuring the optical density (OD) at λ = 570 nm in a spectrophotometer. Data obtained were normalized to controls (non-irradiated hASCs or non-irradiated and non-treated hFFs) and expressed as the mean ± SD of three different experiments performed in quintuplicate.

### 2.6. Measurement of ROS

Intracellular levels of ROS were detected using 2′-7′-dichlorofluorescein diacetate (H2DCF-DA, Molecular Probes, Eugene, OR, USA). In brief, 10^4^ cells/well were seeded in 96-well plates for 24 h, washed with 1× PBS and incubated with H2DCF-DA at a final concentration of 10 μM, at 37 °C for 30 min. After this period, the fluorescent probe was removed and cells were washed with 1× PBS. Finally, cells were exposed to different UVB irradiation doses (10, 20 and 40 mJ/cm^2^) and intracellular ROS accumulation was measured for 1 h in a microplate reader (excitation wavelength [λex] = 490; emission wavelength [λem] = 538 nm). The results obtained were normalized to the cell viability values and expressed as the mean ± SD of at least three independent experiments performed in quintuplicate.

### 2.7. Cytotoxicity Assays

Cytotoxicity was assessed by the quantification of extracellular lactate dehydrogenase (LDH) detected in culture medium. For these assays, 10^4^ cells/well were seeded in 96-well plates. LDH assays were performed according to the manufacturer’s protocol (Cytotoxicity Detection Kit, Roche, Mannheim, Germany). In brief, 24 and 48 h after UVB irradiation, LDH in culture media was measured as an indicator of cytotoxicity as this enzyme is released from damaged cells. The kit reaction mixture added to each well was allowed to react for 30 min in darkness at room temperature (RT); then, the absorbance was measured at λ = 490 nm in the spectrophotometer. Absorbance data were normalized to the value obtained from cells cultured with 1% *v*/*v* Triton X-100 (100% mortality), and cytotoxicity was expressed as a percentage of the value in untreated control cells. The assays were performed at least three times in quintuplicate.

### 2.8. Analysis of Nrf2 Expression by Western Blot

To investigate Nrf2 expression, HC016 cells and hASCs were seeded at a density of 3 × 10^5^ cells/well onto 6-well plates (Corning, Tewksbury, MA, USA) and collected at 1 h after the UVB irradiation. Then, cells were lysed in 1× Laemmli buffer (Sigma-Aldrich, St. Louis, MO, USA) and sonicated to obtain a homogeneous sample. Nuclear extracts were prepared according to a previously described protocol [32]. In brief, protein lysates were boiled for 5 min, subsequently separated on 10% SDS-PAGE and transferred onto a nitrocellulose membrane (GE Healthcare, Life Sciences, Freiburg, Germany). A 5% skimmed milk in Tris-buffered saline with Tween (20 mM Tris, 500 mM NaCl, 0.1% Tween-20 [*v*/*v*], pH 7.5) was used to block the membranes for 1 h and. After blocking, membranes were incubated overnight at 4 °C with anti-Nrf2 primary antibody (1:500) and Lamin A/C (1:5000, Genetex, Irvine, CA, USA). Subsequently, membranes were washed and incubated with the secondary antibody goat anti-rabbit IgG (1:1000, Thermo Fisher Scientific, Waltham, MA, USA) for 1 h at RT. Finally, SuperSignal West Pico PLUS Chemiluminescent Substrate (Thermo Fisher Scientific, Waltham, MA, USA) was used to visualize the membranes and images were recorded with the G:Box Chemi HR16 gel documentation system (Syngene, Frederick, MD, USA). Densitometry analyses were performed with ImageJ sofware (NIH, Bethesda, MD, USA). The data were normalized to those of the corresponding loading controls and were expressed relative to non-UVB exposed hASCs.

### 2.9. Cell Localization of Nrf2 by Immunofluorescence Assay

One hour after UVB exposure, Nrf2 intracellular localization was assessed by immunofluorescence assays. For this experiment, 7 × 10^3^ cells were seeded on 12-mm coverslips (Menzel-Gläser, Waltham, MA, USA). After a 30-min fixation at RT with 4% paraformaldehyde (PFA, PanReac AppliChem, Barcelona, Spain), cells were permeabilized and non-specific protein interactions were blocked by incubation with 1× PBS, containing 3% BSA and 0.1% Triton X-100, for another 30 min at RT. Cells were then incubated overnight at 4 °C with the primary antibody (anti-Nrf2 antibody, 1:500, Genetex, Irvine, CA, USA). After incubation, cells were washed three times with 1× PBS and incubated with a secondary antibody (Goat anti-Rabbit IgG Alexa 488, 1:2000, Invitrogen, Eugene, OR, USA) for 1 h at RT in the dark. Finally, after three more times washes, cells were observed under a Zeiss LSM800 confocal microscope (Carl Zeiss, Chicago, IL, USA) using a ×20 objective.

### 2.10. Fluorescent Labeling of Cytoskeletal Proteins

For this experiment, 3 × 10^4^ hFFs were seeded on 8-well μ-Slides (Ibidi, Martinsried, Germany) and treated with HC016-CM. At 24 h after CM treatment, hFFs were irradiated in PBS and cultivated for 48 h in FBS-free DMEM. The cells were washed in 1× PBS, fixed in 4% PFA, permeabilized in 1% Triton X-100 for 30 min at RT, and then incubated with Phalloidin-iFluor 488 (Abcam, Cambridge, UK) in 3% bovine serum albumin (BSA, Sigma-Aldrich, St. Louis, MO, USA) for 1 h. Nuclei were identified with Hoechst (Abcam, Cambridge, UK). Images were examined under a Zeiss Axioskop fluorescent microscope (Apotome 2, Carl Zeiss, Oberkochen, Germany) using a 40× objective (λex = 493 nm and λem = 517 nm).

### 2.11. Apoptosis Analysis

Apoptosis was determined by flow cytometry using an Alexa Fluor 488 Annexin V/PI Dead Cell Apoptosis Kit (Thermo Fisher Scientific, Waltham, MA, USA). In brief, cells were seeded at a density of 3 × 10^5^ cells/well onto 6-well plates and, 24 h after UVB irradiation the hFFs were harvested and stained with the apoptosis kit for 15 min at RT (1 × 10^6^ cells/mL). Fluorescence of the stained cells were quantified by flow cytometry (λex = 488 and 535 nm; λem = 499 and 617 nm, respectively). Figures were analyzed using Flowing Software (v2.5.1.0, Turku Centre for Biotechnology, University of Turku, Turku, Finland) and results were expressed as the mean ± SD of three independent experiments. Histograms presented are representative of these experiments.

### 2.12. Senescence Evaluation by SA-β-Gal Staining

Senescence-associated beta-galactosidase (SA-β-Gal) activity was performed using a Senescence Histochemical Staining Kit (Sigma-Aldrich, St. Louis, MO, USA). For this experiment, 6.2 × 10^4^ hFFs were seeded in 24-well plates (Sarstedt, Nümbrecht, Germany) and treated with HC016-CM. At 48 h after UVB irradiation, cells were washed in 1× PBS, fixed for 7 min in 0.2% formaldehyde at RT and stained according to the manufacturer’s instructions. The population of blue-stained SA β-Gal positive cells was analyzed using a phase-contrast microscope (Nikon Eclipse TS100, Melville, NY, USA) and data were expressed as the percentage of the total number of cells counted in five random fields on each slide. Images presented are representative of these slides.

### 2.13. Analysis of IL-6 and Collagen Type I Expression by qRT-PCR

To analyze the expression of *IL-6* and collagen type I, 3 × 10^5^ hFFs were seeded in 6-well plates (Corning, Tewksbury, MA, USA) and treated with HC016-CM. At 24 h after UVB irradiation, total cellular RNA was extracted using NucleoSpin^®^ RNA XS Kit (Macherey-Nagel, Düren, Germany) following the manufacturer’s protocol, and the RNA concentration was determined using a NanoDrop ND-1000 spectrophotometer (Thermo Fisher Scientific, Waltham, MA, USA). Then, cDNA was synthesized from 300 ng total RNA using the iScript^TM^ cDNA Synthesis Kit (Bio-Rad, Segrate, MI, Italy).

Amplification reaction assays contained 12.5 ng cDNA, 4 µL SYBER Green Supermix (Bio-Rad, Segrate, MI, Italy) and primers at an optimal concentration in a total volume of 10 µL. The primers are listed in Table 1. qRT-PCR was performed using the CFX96 thermal cycler (Bio-Rad, Segrate, MI, Italy) under the following conditions: a hot start at 93 °C for 3 min followed by 40 cycles (95 °C for 30 s, 55 °C for 30 s and 72 °C for 1 min). Data were analyzed using CFX Manager software (v3.1, Bio-Rad, Segrate, MI, Italy) and *GAPDH* was used as the housekeeping gene. Each sample was tested in triplicate and three independent replicate experiments were performed.

### 2.14. Protein Quantification

IL-6 and collagen type I biosynthesis were measured using a commercial enzyme-linked immunosorbent assay (ELISA) kit (Human IL-6 and Human Pro-Collagen Iα1/COLIA1, R&D Systems, Minneapolis, MN, USA) following the manufacturer’s instructions. In brief, cells were cultured in 6-well plates (3 × 10^5^ cells/well), treated with HC016-CM for 24 h, and exposed to UVB. The concentration of IL-6 and collagen type I in cell culture supernatants was quantified at 24 h after UVB irradiation, and the absorbance at 450 and 540 nm was measured using a microplate spectrophotometer. The results obtained were normalized to the number of cells and expressed as the mean ± SD of at least three independent experiments performed in triplicate.

### 2.15. Cell Migration Assays

Migration assays were performed using a 35-mm dish containing 2-well silicone inserts with a defined cell-free gap (Ibidi, Martinsried, Germany). For this experiment, 7 × 10^3^ cells were seeded in each well and treated for 24 h with HC016-CM. Immediately after UVB irradiation, the silicone gasket was carefully removed and cultures were maintained in FBS-free DMEM for 24 and 48 h. To record wound closure, images were captured at 0, 24 and 48 h time points using a phase-contrast microscope. Cell migration was quantified by counting the number of cells that migrated to the gap area. Results were normalized to controls (non-irradiated hASCs or non-irradiated non-treated hFFs) and expressed as the mean ± SD of three independent experiments. Images presented are representative of these experiments.

### 2.16. Statistical Analysis

The number of replicates analyzed is reported for each experiment. All data are presented as mean ± SD. For statistical analysis, Prism^®^ statistical v5.0 software (GraphPad Software, San Diego, CA, USA) was used. The results were verified for normality by the Kolmogorov–Smirnov test. According to the design of each experiment, either analysis of variance followed by Bonferroni post hoc test for multiple comparison between groups or two-tailed Student’s *t*-test was performed. A confidence level of 95% was established for all cases.

## 3. Results

### 3.1. Deleterious Effect of UVB Irradiation on hFFs

**Figure 1:**
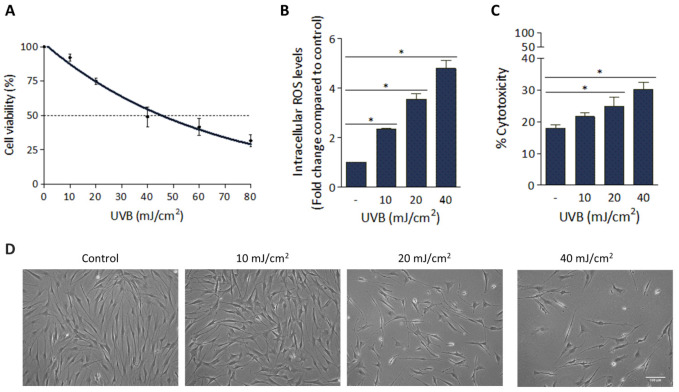
Dose-dependent effect of UVB radiation on hFFs. (**A**) Cells were exposed to 0–80 mJ/cm^2^ UVB radiation, and the percentage of viable cells was measured 48 h after exposure. (**B**) ROS generation was induced by UVB radiation (10, 20 and 40 mJ/cm^2^) and measured at 1 h. Results were normalized to controls (non-irradiated cells). (**C**) Percentage of cytotoxicity as measured by LDH levels in the culture medium 48 h post-irradiation. (**D**) Phase-contrast images of HFFs 48 h after UVB irradiation (scale bar: 100 µm). Data are expressed as mean ± SD. * *p* < 0.05 compared to controls (non-irradiated cells).

The harmful effect of UVB radiation on hFFs was analyzed by assessing cell viability, the intracellular ROS levels, the amount of LDH released from cells to the cultured media as an indicator of the cytotoxic effect and cell morphological changes. For cell viability analysis, the cultured hFFs were exposed to a wide range of UVB irradiation doses (0 to 80 mJ/cm^2^). As can be seen in Figure 1A, a clear dose-dependent cell number reduction (*R*^2^ = 0.96) was observed. For the following experiments, we selected the doses of 10, 20 and 40 mJ/cm^2^, all of them below the LD_50_ of 45.7 mJ/cm^2^. Measuring intracellular ROS levels 1 h after UVB irradiation and comparing them to values in non-irradiated cells, significant increases were registered (2.3, 3.5 and 4.8-fold with doses of 10, 20 and 40 mJ/cm^2^, respectively; *p* < 0.001; Figure 1B). Similarly, dose-dependent increases in LDH levels were noted, the estimated percentage of cytotoxicity of hFFs at 48 h post-irradiation being 21.6 ± 1.1%, 24.7 ± 3.0% and 30.1 ± 2.3% with doses of 10, 20 and 40 mJ/cm^2^, respectively (*p* < 0.05; Figure 1C). Finally, UVB-induced morphological changes were also analyzed at 48 h. As shown in Figure 1D, with the lowest dose (10 mJ/cm^2^) hFFs remained adhered to the substrate and cells showed typical cytoplasmic elongation; however, when exposed to 20 or 40 mJ/cm^2^, in addition to a reduction in the cell population, it was observed that cells lost their usual elongated morphology and appeared rounded, a sign of hFF culture damage.

### 3.2. H_2_O_2_-Preconditioning Reduces UVB-Induced Damage in hASCs

**Figure 2:**
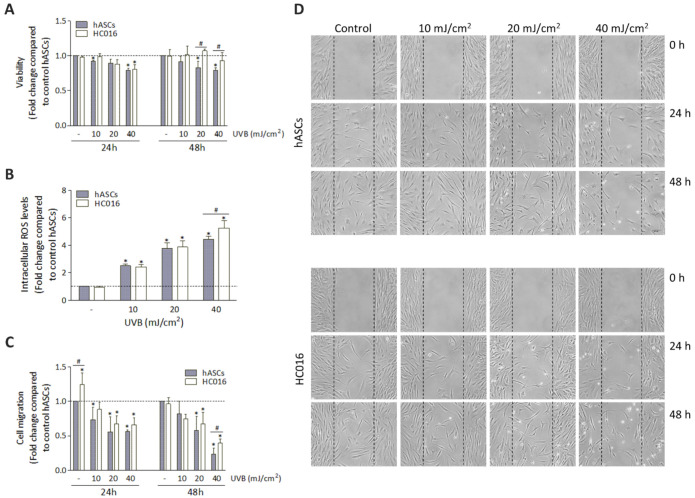
Effect of H_2_O_2_-preconditioning on hASC response to UVB radiation. (**A**) Viability of hASCs and HC016 cells, 24 and 48 h after UVB exposure. (**B**) ROS generation was induced by UVB radiation and measured by H2DCF-DA staining at 1 h. (**C**) Quantitative data on cell migration: fold difference in cell count. (**D**) Representative photomicrographs of cell migration at 0, 24 and 48 h after irradiation. The dashed lines indicate the wound edges (scale bar: 50 µm). Values were normalized to non-irradiated control hASCs (dotted line) and expressed as mean ± SD. * *p* < 0.05 with respect to the control of non-irradiated hASCs; # *p* < 0.05 between hASCs and HC016 cells.

To evaluate the cytoprotective effect of the H_2_O_2_-preconditioning against the deleterious effects of UVB, hASCs and HC016 cells were also exposed to 10, 20 and 40 mJ/cm^2^ of UVB radiation, and viability, intracellular ROS levels and cell migration were measured. Firstly, compared to non-irradiated hASCs, UVB irradiation was followed by a decrease in viability in both cell populations in a dose-dependent manner at 24 h; however, at 48 h, only the HC016 cells had recovered their viability, reaching values close to those of the non-irradiated controls. Specifically, at this point, the viability of HC016 cells was 1.3- and 1.2-fold higher than that of hASCs with doses of 20 and 40 mJ/cm^2^, respectively (*p* < 0.05; Figure 2A).

Secondly, when analyzing intracellular ROS levels at 1 h after UVB irradiation, a dose-dependent increase was observed in both cell types (Figure 2B), and significant differences between preconditioned and non-preconditioned cells were only found with the irradiation dose of 40 mJ/cm^2^ (1.2-fold higher in HC016 cells; *p* < 0.01).

Thirdly, concerning cell migration, one of the fundamental properties of hASCs from the therapeutic point of view, non-irradiated HC016 control cells initially showed a better migration ability (1.2-fold higher than that of the control hASCs at 24 h; *p* < 0.05), but this effect was not maintained at 48 h. After UVB irradiation, dose-dependent reductions in migration capacity were observed in both cell populations, but when the highest irradiation dose was used, HC016 cells showed greater cell migration than hASCs (1.2-fold more cells at 48 h; *p* < 0.05; Figure 2C,D).

### 3.3. H_2_O_2_-Preconditioning Induces Nrf2 Expression in Response to UVB Exposure

**Figure 3:**
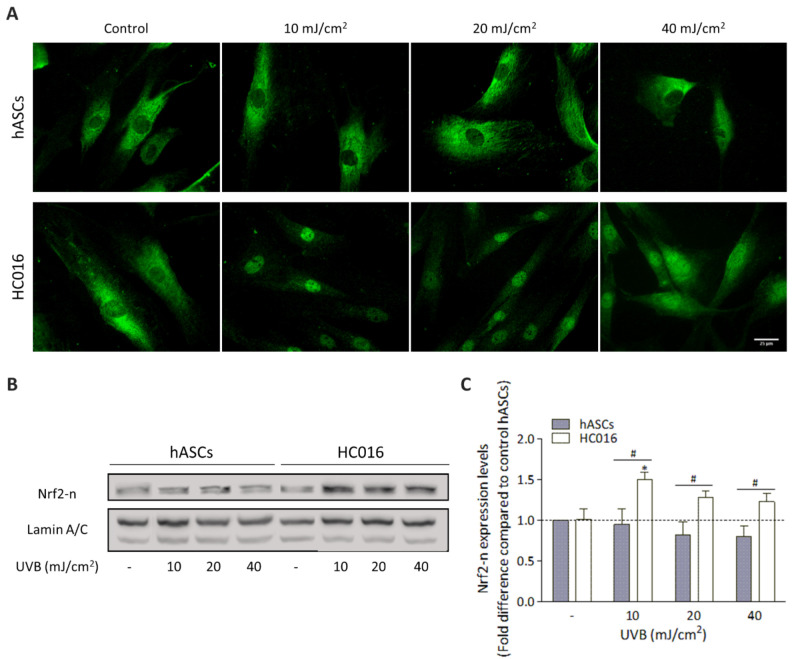
H_2_O_2_-preconditioning increases the expression of Nrf2-n. (**A**) Representative images of Nrf2 intracellular localization evaluated by immunofluorescence (scale bar: 25 µm). (**B**) Western blot analysis of Nrf2-n expression and (**C**) optical density quantification. Values were normalized to non-irradiated control hASCs (dotted line) and expressed as mean ± SD. * *p* < 0.01 with respect to the control of non-irradiated hASCs; # *p* <0.05 between hASCs and HC016 cells.

To analyze the effect of H_2_O_2_-preconditioning on the hASC antioxidant response to UVB irradiation, the expression of Nrf2 was studied. As seen in Figure 3A, in non-irradiated cells, Nrf2 was located in the cytoplasm in both hASCs and HC016 cells; however, 1 h after UVB exposure, H_2_O_2_-preconditioned cells exhibited Nrf2 nuclear (Nrf2-n) translocation. In fact, at this point, Nrf2-n expression was significantly higher in HC016 cells (Figure 3B), being 1.6-, 1.6- and 1.5-fold higher than in hASCs with irradiation doses of 10, 20 and 40 mJ/cm^2^, respectively (*p* < 0.05; Figure 3C).

### 3.4. Effect of HC016-CM Treatment on hFFs

#### 3.4.1. HC016-CM Does Not Alter hFF Basic Cell Culture Parameters

Given that paracrine activity is one of the main mechanisms through which MSCs exert their therapeutic effects, we first evaluated the effect of HC016-CM treatment on hFFs under standard conditions (without UVB irradiation). In particular, proliferation rate, intracellular ROS levels, cytotoxicity and cell morphology were analyzed.

**Figure 4:**
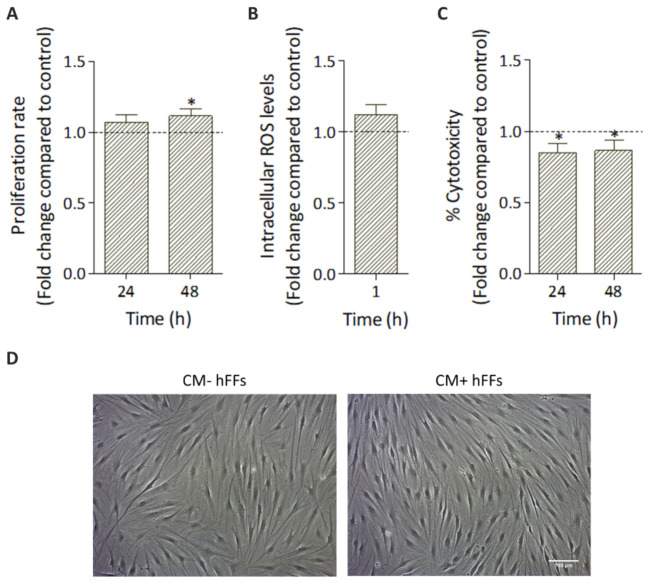
Effect of HC016-CM treatment on hFFs under standard conditions (without UVB irradiation). (**A**) Proliferation rate at 24 and 48 h post-treatment. (**B**) ROS levels measured at 1 h. (**C**) Percentage of cytotoxicity as measured by LDH levels in the culture medium 24 and 48 h post-treatment. (**D**) Phase-contrast images of hFFs 48 h after UVB irradiation (scale bar: 100 µm). Values were normalized to non-treated cells (dotted line) and expressed as mean ± SD. * *p* < 0.01 compared with non-treated cells.

Cell proliferation rate was slightly, but significantly, higher in HC016-CM-treated cells (1.1-fold higher than in non-treated hFF cultures at 48 h; *p* < 0.01; Figure 4A). Similarly, a small augmentation in ROS levels was also observed in treated hFFs, but, in this case, the difference was not statistically significant (*p* > 0.05; Figure 4B). In addition, the percentage of cytotoxicity registered in treated hFF cultures was 1.2-fold lower than in the controls (*p* < 0.01; Figure 4C). Finally, as shown in Figure 4D, no differences in in vitro cell morphology were found between treated and non-treated cells.

#### 3.4.2. HC016-CM Protects hFFs against UVB-Induced Damage

To evaluate the therapeutic potential of HC016-CM to protect hFFs against UVB-induced damage, before UVB irradiation, hFFs were cultured for 24 h with HC016-CM and cellular viability, intracellular ROS levels, cytotoxicity, apoptosis, cell morphology and senescence were analyzed.

**Figure 5:**
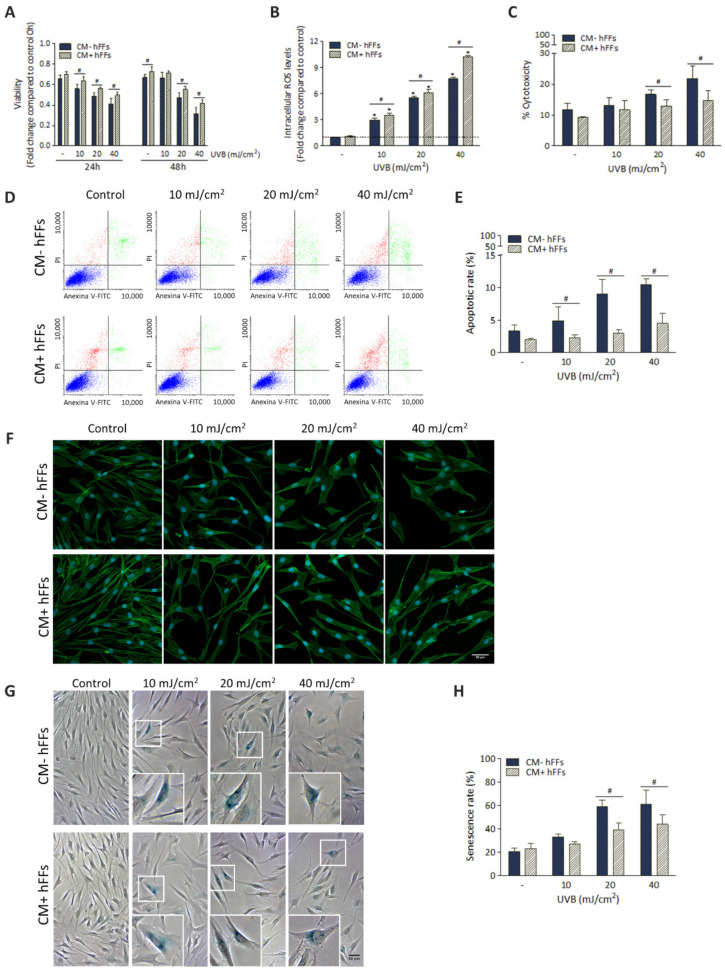
The photoprotective effect of HC016-CM on hFFs exposed to UVB radiation. (**A**) The viability of CM− and CM+ hFFs was measured at 24 and 48 h after UVB exposure and results were standardized relative to values of non-irradiated non-treated cells at time 0 h. (**B**) ROS levels were measured at 1 h and results were normalized to non-irradiated CM− hFFs. (**C**) Percentage of cytotoxicity as measured by LDH levels in the culture medium 48 h post-irradiation. (**D**) Representative graphs of annexin V/PI assay performed by flow cytometry at 24 h. Annexin V−/PI− stains live cells (blue), annexin V+/PI− or V+/PI− early or late apoptosis respectively (green) and annexin V−/PI+ necrosis (red). (**E**) Quantification of cells undergoing early and late apoptosis. (**F**) Representative fluorescence images of F-actin cytoskeleton obtained at 48 h post-irradiation (scale bar: 50 µm). Images show F-actin filaments stained with green fluorescent Alexa iFluor 488-conjugated phalloidin, and nuclei stained with Hoechst (blue). (**G**) SA-β-gal staining in CM− and CM+ hFFs 48 h after UVB irradiation. Representative phase-contrast images (scale bar: 50 µm). Senescent cells are identified by the blue staining of the cytoplasm due to SA-β-gal activity. (**H**) Percentage of SA-β-gal positive cells out of the total number of cells counted. Data are expressed as mean ± SD. * *p* < 0.05 compared with non-irradiated CM− hFFs; # *p* < 0.05 between HC016-CM-treated hFFs and untreated cells.

HC016-CM treatment was associated with significantly greater hFF viability, this effect being more evident with the highest doses of irradiation. Specifically, we found that, at 24 h, the viability of hFFs treated with HC016-CM was 1.1-, 1.1- and 1.2-fold higher than that of non-treated cells with doses of 10, 20 and 40 mJ/cm^2^, respectively (*p* < 0.05), and at 48 h 1.2- and 1.3-fold higher with doses of 20 and 40 mJ/cm^2^, respectively (*p* < 0.05; Figure 5A). Regarding ROS generation, curiously, cells treated with HC016-CM showed significantly higher intracellular ROS levels (1.2-, 1.1- and 1.3-fold higher than those in non-treated cells with doses of 10, 20 and 40 mJ/cm^2^, respectively, 1 h after UVB irradiation; *p* < 0.05; Figure 5B).

Notably, HC016-CM treatment was associated with significantly less release of LDH to the culture medium (1.3- and 1.5-fold lower levels with doses of 20 and 40 mJ/cm^2^, respectively, at 48 h after UVB irradiation; *p* < 0.05; Figure 5C). Similarly, the percentage of apoptotic cells was significantly lower in HC016-CM treated than in untreated cultures (Figure 5D). In particular, in the case of cultures exposed to 10, 20 and 40 mJ/cm^2^, the percentages of apoptotic cells were 2.1-fold (2.3 ± 0.4% in hFFs treated with HC016-CM vs. 4.9 ± 2.1% in untreated hFFs), 3.0-fold (3.0 ± 0.6% vs. 9.0 ± 2.3%) and 2.3-fold (4.5 ± 1.5% vs. 10.4 ± 0.9%; *p* < 0.01; Figure 5E) lower.

As shown in Figure 5F, HC016-CM treatment was associated with an attenuation of the UVB-induced alterations in cell morphology, such as cell widening and flattening seen in untreated cells. Moreover, cell staining with the specific senescence-associated marker β-Gal (SA-β-gal) allowed us to observe that, at 48 h, there were also significantly lower percentages of SA-β-Gal-positive cells in HC016-CM-treated than in untreated cultures (39.0 ± 6.0% vs. 59.0 ± 5.7%, and 43.9 ± 7.8% vs. 61.2 ± 11.8%, with doses of 20 mJ/cm^2^ and 40 mJ/cm^2^, respectively; Figure 5G,H).

### 3.5. HC016-CM Treatment Reduces UVB-Induced Alterations in mRNA Expression and Synthesis of IL-6 and Pro-Collagen Type I in hFFs

We next investigated the UVB-induced modifications in mRNA expression and protein synthesis of IL-6 and pro-collagen type I in hFFs, and whether HC016-CM treatment was able to attenuate these undesirable effects. For this, qRT-PCR was performed 24 h after UVB irradiation to determine gene expression and ELISA analysis to quantify protein synthesis.

**Figure 6:**
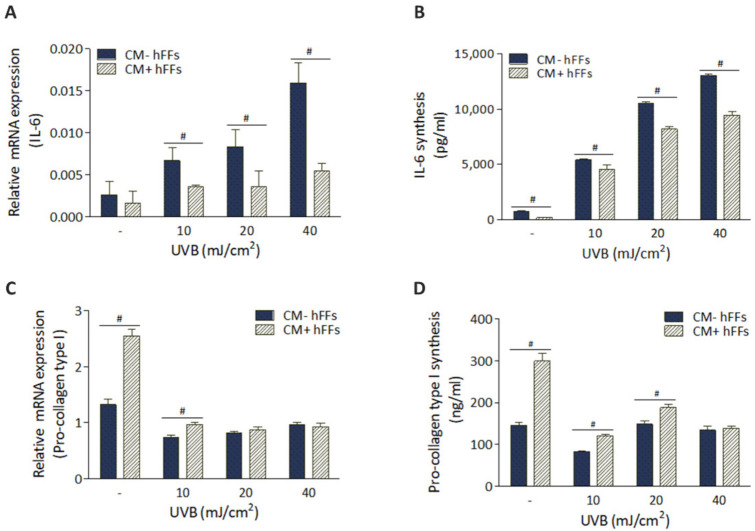
HC016-CM treatment reduces the expression of UVB-induced *IL-6* and promotes the expression of pro-collagen type I in hFFs. Reverse transcription-quantitative polymerase chain reaction (qPCR) analysis of (**A**) *IL-6* and (**C**) pro-collagen I at 24 h post-UVB irradiation. Biosynthesis of (**B**) IL-6 and (**D**) pro-collagen type I measured by ELISA 24 h after irradiation. Data are expressed as mean ± SD. # *p* < 0.05.

As expected, UVB radiation was associated with significant increases in the expression and synthesis of IL-6 in hFFs in a dose-dependent manner, this effect being partially attenuated after HC016-CM treatment. Specifically, 24 h after UVB irradiation with doses of 10, 20 and 40 mJ/cm^2^, *IL-6* expression levels were 1.9-, 2.3- and 2.9-fold lower in treated than in untreated hFFs (*p* < 0.05; Figure 6A), while IL-6 synthesis was 1.2-, 1.3- and 1.4-fold lower, respectively (*p* < 0.001; Figure 6B). It should be noted that this effect on IL-6 secretion related to HC016-CM treatment was also observed in non-irradiated hFFs (3.7-fold lower levels than in non-treated HFFs, *p* < 0.05; Figure 6B).

In the case of pro-collagen type I, as seen in Figure 6C,D, in the absence of UVB irradiation, the mRNA expression of pro-collagen type I and its synthesis were higher in HC016-CM treated cells (1.9- and 2.0-fold higher than in non-treated hFFs, respectively, *p* < 0.001). Following UVB irradiation, the mRNA expression in cultures treated with HC016-CM was 1.4-fold higher than in untreated hFFs with the dose of 10 mJ/cm^2^ (*p* < 0.001), while its synthesis was 1.4- and 1.3-fold higher with doses of 10 and 20 mJ/cm^2^, respectively (*p* < 0.001).

### 3.6. HC016-CM Treatment Attenuates the Deleterious Effect of UVB Radiation on hFF Migration

**Figure 7:**
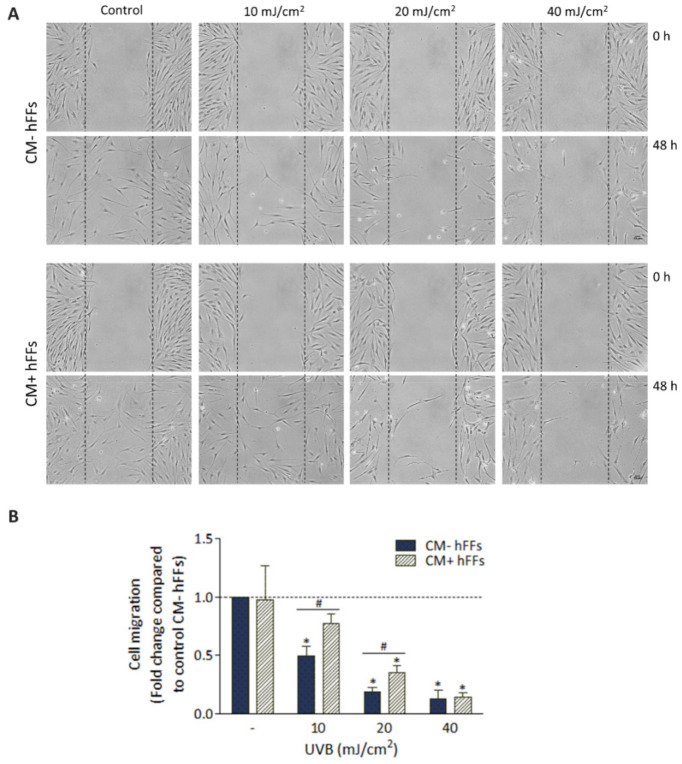
Effect of HC016-CM on wound closure. (**A**) Representative photomicrographs of wounded hFFs and cell migration at 0 and 48 h after irradiation. The dashed lines indicate the wound edges (scale bar: 50 µm). (**B**) Quantitative data on cell migration 48 h after UVB exposure: fold difference in cell count. Results were normalized to controls (non-irradiated cells, dotted line). Data are expressed as mean ± SD. # *p* < 0.01. * *p* < 0.05.

Finally, to investigate the effect of HC016-CM on the migratory response of hFFs, a wound-healing assay was performed. After obtaining the 48-h time point photomicrographs, we first checked that HC016-CM treatment did not modify the migratory capacity of the non-irradiated hFFs. Second, we noted that despite UVB irradiation being associated with dose-dependent reductions in cell migration in both treated and non-treated hFFs (Figure 7A), treated hFFs showed greater wound repair potential. In particular, the migratory capacity of hFF cultures irradiated with doses of 10 and 20 mJ/cm^2^, and treated with HC016-CM, was 1.6- and 1.8-fold higher respectively than in the corresponding controls (*p* < 0.05; Figure 7B).

## 4. Discussion

There is considerable evidence that UVB radiation induces cellular damage through oxidative stress, peroxidation of DNA, proteins, and lipids and inflammation, factors that have been associated with skin aging, mutagenesis and photocarcinogenesis [10,34]. In this context, cell therapy with MSCs is emerging as a promising alternative for the prevention and/or treatment of skin photodamage. MSCs are able to induce the regeneration of dermal cells, through their paracrine activity, stimulating angiogenesis and protecting these cells against oxidative stress [20,35].

Firstly, analyzed UVB-induced damage in an in vitro UVB irradiation model in cultured hFFs. In irradiated dermal fibroblasts, intracellular ROS increased in a dose-dependent manner, resulting in reduced proliferation and increased cytotoxicity. In addition, with doses of 20 and 40 mJ/cm^2^, cells lost their usual elongated morphology and appeared rounded, a sign of damage to the cultured hFFs.

Secondly, to enhance the therapeutic properties of hASCs, and using our previous studies as a reference, we assessed the effect of H_2_O_2_-preconditioning on hASCs in response to UVB radiation. At 1 h after irradiation, ROS levels increased in a dose-dependent manner in both hASCs and HC016 cells; however, the levels were higher in HC016 cells that had received the highest dose of irradiation (40 mJ/cm^2^). This result is consistent with a previous finding by our research group, according to which hASCs limit their mitochondrial activity in an attempt to reduce intracellular ROS levels, while HC016 cells increase mitochondrial activity since they have a greater concentration of antioxidants that can counteract ROS [32]. Therefore, the observed increase in ROS levels could be a consequence of a transient increase in oxidative phosphorylation of HC016 cells to obtain ATP more quickly and survive in a hostile microenvironment [36]. It must be emphasized that, while high levels of ROS can damage DNA, proteins and lipids, moderate levels of ROS are necessary to activate essential signaling pathways that regulate cell functions such as proliferation and differentiation [37,38,39]. In line with this, we observed that both hASCs and HC016 cells had become less viable by 24 h after irradiation; however, at 48 h, only HC016 cells recovered their viability, reaching values similar to those of the non-irradiated control hASCs, even with the highest irradiation dose.

The enhanced survival of HC016 cells is likely due to the activation of different signaling pathways, and since our studies are based on an oxidative stress model, we evaluated the Nrf2-ARE signaling pathway, an important cellular mechanism for reducing oxidative stress [14,15,40]. Before irradiation, Nrf2 was located in the cytoplasm in both hASCs and HC016 cells; however, 1 h after UVB exposure, H_2_O_2_-preconditioned cells exhibited Nrf2 translocation to the nucleus together with an increase in its expression. As we have previously shown, the overexpression of Nrf2 leads to increases in levels of heme oxygenase-1 (HO-1), superoxide dismutase-1 (SOD-1), glutathione peroxidase-1 (GPx-1) and catalase (CAT) which can neutralize increases in ROS levels and thus enhance the survival of HC016 cells [32,41].

On the other hand, since one of the main mechanisms underlying the therapeutic potential of MSCs is their ability to migrate to the site of injury [23], we analyzed the effect of hASC preconditioning on their migration capacity after UVB irradiation. With the highest radiation dose (40 mJ/cm^2^), this migratory ability was also greater in the HC016 (1.7-fold higher) than in the non-preconditioned cells. These results are consistent with the results of other studies showing increases in the migratory capacity of H_2_O_2_-preconditioned MSCs under oxidative stress conditions by increasing the expression of stromal cell-derived factor 1 (SDF-1) and its receptor CXCR4, both involved in the process of activating cell migration and survival in damaged tissues [42,43].

As mentioned above, previous studies have demonstrated that the therapeutic potential of hASCs is not only attributable to their abilities to differentiate and migrate to the site of injury, but also to the factors they secrete [44,45]. Palomares et al. showed that the antioxidant capacity of hASCs-CM is 1.8-fold higher than that of standard culture medium [46]. Hence, thirdly, given that preconditioned cells have a greater capacity to respond to oxidative stress, we tested HC016-CM as a photoprotective therapy for hFFs. For this, prior to UVB irradiation, hFFs were cultured for 24 h with HC016-CM. Before the subsequent steps, we checked that HC016-CM did not affect hFF morphology or intracellular ROS levels under standard conditions, and only observed a reduction in the percentage of cytotoxicity together with a slight increase in their proliferative capacity, a response also described by other authors [47]. Then, we evaluated the therapeutic potential of HC016-CM treatment to overcome the deleterious effect of UVB radiation in hFFs. Results showed a clear photoprotective effect of HC016-CM, evidenced by greater viability, a lower activity of the LDH released into the extracellular medium and a lower percentage of apoptosis in treated than in non-treated hFFs. These effects were most evident with the higher doses of irradiation (20 and 40 mJ/cm^2^). The cytoprotective effect could be due, among other reasons, to the presence in HC016-CM of, on the one hand, various growth factors with mitosis-inducing effects on quiescent cells [48], and on the other hand, apoptosis-inhibiting proteins such as Bcl-2, survivin and Akt [49].

Regarding ROS generation, curiously, intracellular ROS levels increased significantly in cells treated with HC016-CM. It must be considered that the presence of an optimal redox level is necessary for the maintenance of cellular homeostasis and that this redox state changes according to metabolic activity and the cellular compartment in which it is carried out [38]. Therefore, a moderate increase in ROS levels can act as a molecular signal to activate signaling pathways involved in cell proliferation and differentiation. In line with this, Liu et al. showed that a moderate increase in ROS levels promotes the proliferation and migration of murine preadipocytes through the regulation of NF-kB p65 [50]; also, Huo et al. reported that increased ROS levels mediate cell adhesion and migration in corneal wound healing [51]. Conversely, an excessive increase in ROS levels can cause oxidative damage that induces senescence and cell death [52]. Nonetheless, in addition to the ROS levels present in the cell, we must also consider the requirement of these levels for various biological activities to be performed at a given time and its mechanisms for intracellular redox regulation.

Thus, the larger increase in ROS levels registered in treated hFFs compared to that in the controls after UVB radiation could be necessary to promote cell migration or reduce senescence; in fact, both effects together with the attenuation of UVB-induced alterations in cell morphology were associated with HC016-CM treatment. In addition, it has been described that senescent cells secrete molecules that influence the cellular and tissue microenvironment, such as the pro-inflammatory cytokines IL-1, IL-6 and IL-8 [53,54]. In line with this, most of the diseases associated with photodamage share characteristics related to inflammation, such as elevated levels of TNF-α and IL-6 [55]. According to other authors, the increased expression of *IL-6* in dermal fibroblasts activates the NF-kB signaling pathway and, consequently, gives rise to an increase in the expression of *MMP-1* and the inhibition of collagen synthesis [6,56]. Moreover, increases in the expression of collagenase and stromelysin and decreases in the expression of inhibitors of MMPs 1 and 3 (*TIMP-1* and *-3*) have been observed in senescent human fibroblasts, which leads to extracellular matrix (ECM) degradation [57]. Conversely, migration, proliferation and ECM remodeling through the synthesis of collagen by fibroblasts are essential in the regeneration of the functional dermis. In our study, we found that UVB radiation induces a dose-dependent increase in the expression and synthesis of IL-6, although this increase was notably lower in treated hFFs. Further, the reduction of collagen expression and synthesis induced by UVB radiation in hFFs was attenuated by the treatment with HC016-CM. Curiously, we also found that, under basal conditions (without irradiation), this treatment increased the expression and synthesis of pro-collagen type I. This is in accordance with the results previously described by Kim et al., in which fibroblasts co-cultured with hASCs in the absence of oxidative stress increased type I collagen synthesis [44].

Finally, we analyzed the effect HC016-CM treatment on hFF migratory capacity. In concordance with the increase observed in pro-collagen type I expression and synthesis, HC016-CM treatment did indeed attenuate the inhibitory effect induced by UVB radiation on the migratory activity of hFFs.

In summary, this study shows that HC016-CM treatment exerts a cytoprotective effect on hFFs against UVB-induced damage, suggesting that the paracrine activity of H_2_O_2_-preconditioned hASCs may be a promising therapy in the prevention of photodamage.

## 5. Conclusions

This study showed that HC016 cells were able to respond efficiently to UVB-induced oxidative stress, which was related to higher Nrf2 antioxidant system activity. Further, HC016-CM treatment prevented damage associated to UVB radiation in hFFs, by reducing cytotoxicity, apoptosis, senescence and IL-6 secretion. Collectively, these findings support the view that HC016 cells could protect against UVB-induced photodamage via paracrine mechanisms.

## Figures and Tables

**Table 1:** Primer sequences.

Gene	Accession Number	Primer Sequence (5′-3′)
*IL-6*	NM_000600	Fw: GCAGATGAGTACAAAAGTCCTGA Rv: TTCTGTGCCTGCAGCTTC
*COL1A2*	NM_000089	Fw: GTGAGAGAGGAGTTGTTGGAC Rv: CCTTCAATCCATCCAGACCAT
*GAPDH*	NM_002046	Fw: ACATCGCTCAGACACCATG Rv: TGTAGTTGAGGTCAATGAAGGG

Abbreviations: *IL-6*, interleukin-6; *COL1A2*, collagen type I alpha 2; *GAPDH*, glyceraldehyde-3-phosphate dehydrogenase.

## Data Availability

The data presented in this study are available in the article.
